# Successful Extensive Pulsed Field Ablation for Atrial Fibrillation Without Leadless Pacemaker Interference

**DOI:** 10.1002/joa3.70457

**Published:** 2026-09-16

**Authors:** Gen Matsuura, Hidehira Fukaya, Tomoharu Yoshizawa, Atsuhiko Sugimoto, Junya Ako

**Affiliations:** ^1^ Department of Cardiovascular Medicine Sagamihara Kyodo Hospital Sagamihara Japan; ^2^ Department of Cardiovascular Medicine Kitasato University School of Medicine Sagamihara Kanagawa Japan

**Keywords:** atrial fibrillation, electromagnetic interference, leadless pacemaker, persistent left superior vena cava, pulsed field ablation

## Abstract

Although Pulsed field ablation (PFA) has demonstrated efficacy for atrial fibrillation, its safety in patients with leadless pacemaker remains uncertain. This case suggests that extensive PFA involving persistent left superior vena cava, superior vena cava, and left atrial posterior wall can be safely performed without device interference.

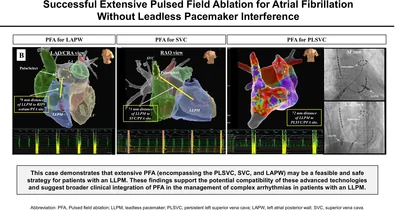

A 75‐year‐old woman was referred to our hospital for symptomatic recurrences of paroxysmal atrial fibrillation (AF) associated with bradycardia‐tachycardia syndrome (BTS). She had previously undergone radiofrequency catheter ablation for AF twice. A leadless pacemaker (LLPM; Micra AV, Medtronic, Minneapolis, MN, USA) had been implanted for BTS (Figure 1A–D), and catheter ablation for AF was planned 5 months later. Preprocedural computed tomography and coronary sinus (CS) angiography revealed the persistent left superior vena cava (PLSVC) (Figure 1E,F).

**FIGURE 1:**
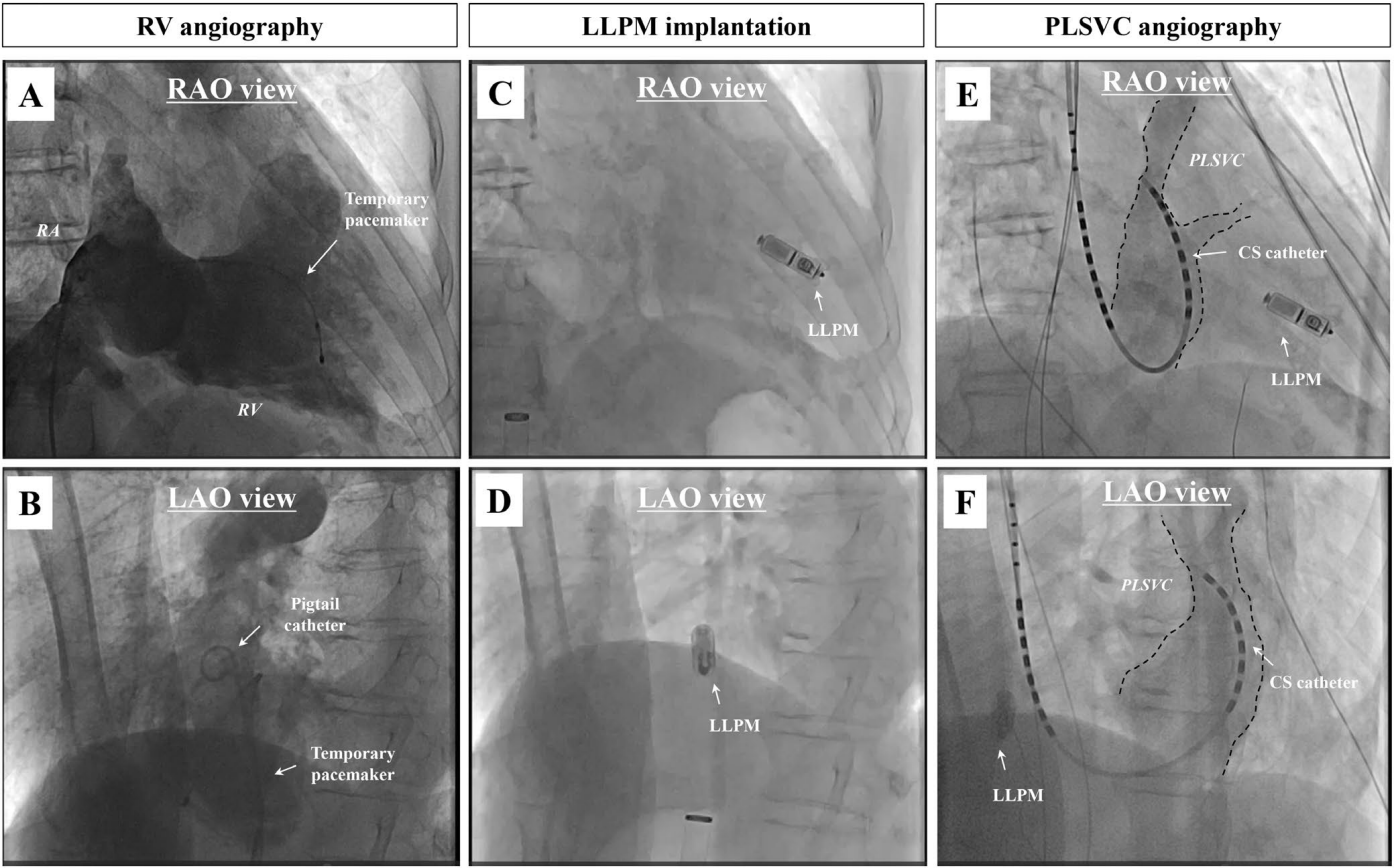
Procedural imaging of LLPM implantation and PLSVC. (A, B) Right ventriculography. (C, D) Fluoroscopy after LLPM implantation. (E, F) CS angiography demonstrating a PLSVC draining into a dilated CS. CS, Coronary sinus; LLPM, Leadless pacemaker; PLSVC, Persistent left superior vena cava.

The procedure was performed under general anesthesia. Transseptal puncture was achieved under intracardiac echocardiographic guidance, and intravenous heparin was administered to maintain an activated clotting time above 350 s. The baseline pacing burden was 6% under the setting of 50 ppm. Before the procedure, the LLPM was programmed to VVI mode at a lower of 50 ppm. During the index procedure, it was changed to VVI 80 ppm during ablation to facilitate detection of Pulsed field ablation (PFA)‐induced electromagnetic interference (EMI). Continuous 12‐lead ECG and intracardiac EGMs were monitored during each PFA application to detect EMI such as the absence of oversensing, inappropriate pacing inhibition, or unexpected mode switching. A voltage map of the left atrium (LA) was created using the EnSite system (Abbott, St. Paul, MN, USA), confirming durable isolation of all four pulmonary veins (PVs). Voltage mapping revealed a low‐voltage area on the LA posterior wall (LAPW). Because the PVs remained isolated despite recurrent AF, the LAPW was selected as an additional substrate‐ablation target. PFA of the LAPW was performed using the PulseSelect catheter under guidance with the EnSite system and fluoroscopy guidance with a total of 77 applications (Figure 2D). The distance between the device and the right inferior PV ostium was 79 mm (Figure 2A,B), and no clinically apparent device interference was observed (Figure 2C). High‐density mapping of the superior vena cava (SVC) identified a myocardial sleeve with local electrograms; therefore, PFA was performed with two applications for potential non‐PV triggers (Figure 3A–C). The distance between the LLPM and the SVC ablation site was 73 mm (Figure 3D), and no device interference was observed (Figure 3E). In addition, spontaneous and catheter‐induced premature atrial contractions arising from the PLSVC reproducibly initiated AF, prompting additional ablation of the PLSVC (Figure 4A,B). To prevent coronary artery vasospasm (CAV), intravenous nitrate (isosorbide dinitrate 1 mg) was administered before delivering five PFA applications. Continuous 12‐lead ECG showed no ST‐segment changes, suggestive of clinically significant CAV; however, coronary angiography was not performed, and CAV could not be definitively excluded. Because general anesthesia precluded intraprocedural phrenic nerve monitoring, diaphragmatic motion was assessed fluoroscopically after the procedure and was preserved. Successful PLSVC ablation was achieved without any device interference (Figure 4C,D). The minimum distance between the LLPM and the PLSVC was 72 mm. The procedure was completed without complications. Post‐ablation mapping confirmed the complete abolition of local electrograms within the LAPW, SVC, and PLSVC. Pre‐ and post‐procedural LLPM parameters were as follows: pacing threshold: 0.38 V and 0.38 V/0.24 ms, R‐wave sensing: 14.6 and 18.5 mV, and impedance: 650 and 700 Ω, respectively. Battery status was unchanged. During the 1‐month follow‐up, there was no evidence of AF recurrence or device malfunction.

**FIGURE 2:**
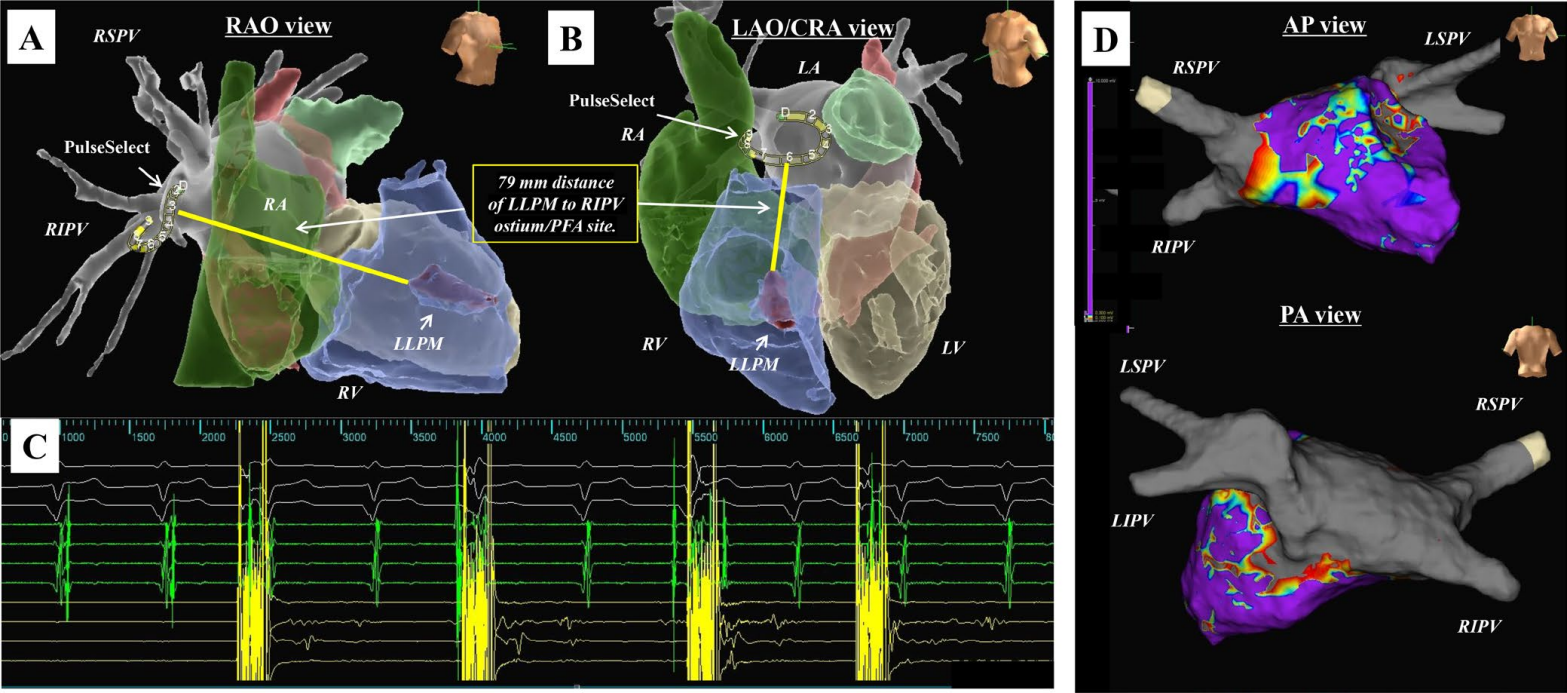
PFA for the LAPW with LLPM. (A, B) The distance between the LLPM and the RIPV was 79 mm. (C) No device interference was observed during PFA for LAPW. (D) Post‐ablation voltage map showing complete LAPW isolation. LAPW, Left atrial posterior wall; LIPV, Left inferior pulmonary vein; LSPV, Left superior pulmonary vein; RIPV, Right inferior pulmonary vein; RSPV, Right superior pulmonary vein.

**FIGURE 3:**
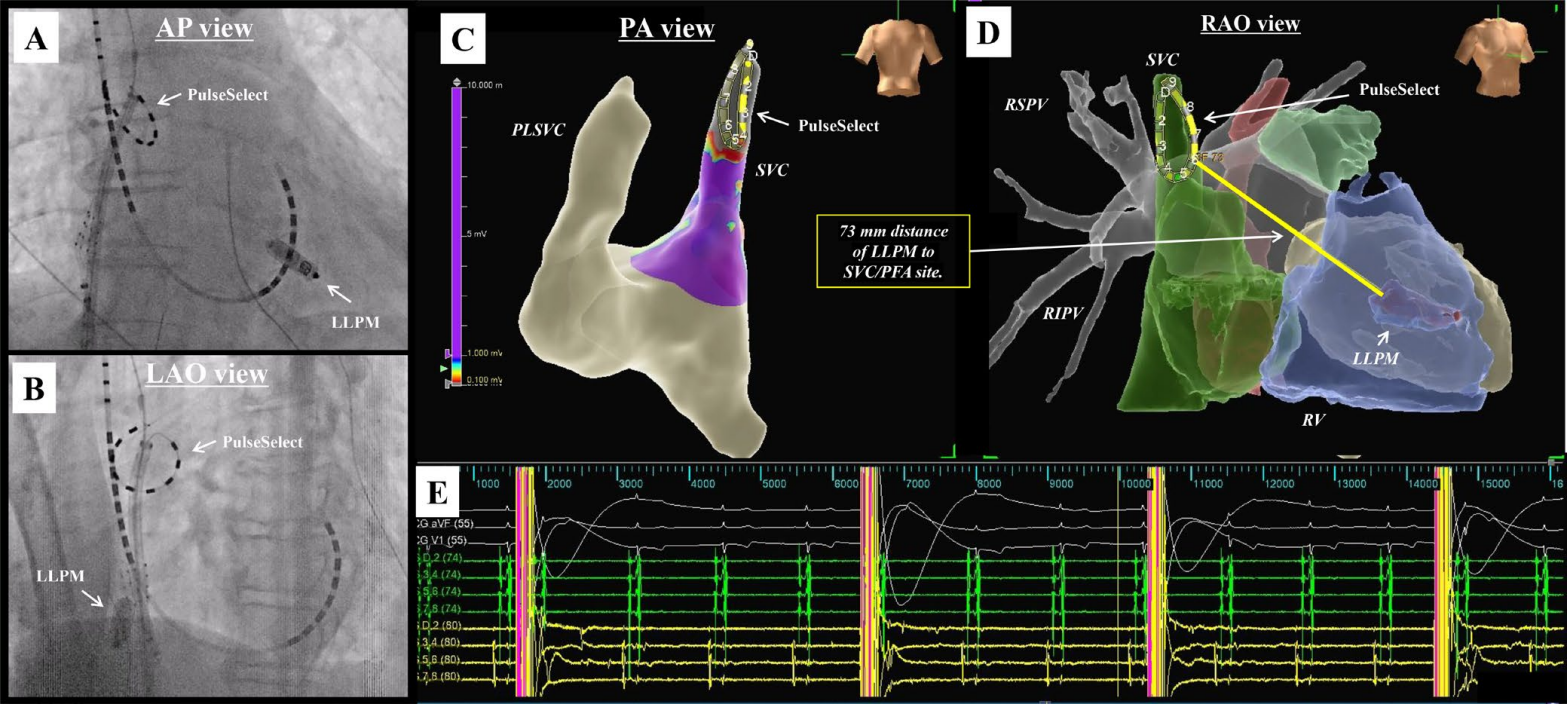
PFA for the SVC with LLPM. (A, B) Fluoroscopic images. (C) Post‐ablation voltage map showing successful SVC isolation. (D) The distance between the LLPM and SVC ablation site was 73 mm. (E) No device interference was observed during SVC ablation. SVC, Superior vena cava.

**FIGURE 4:**
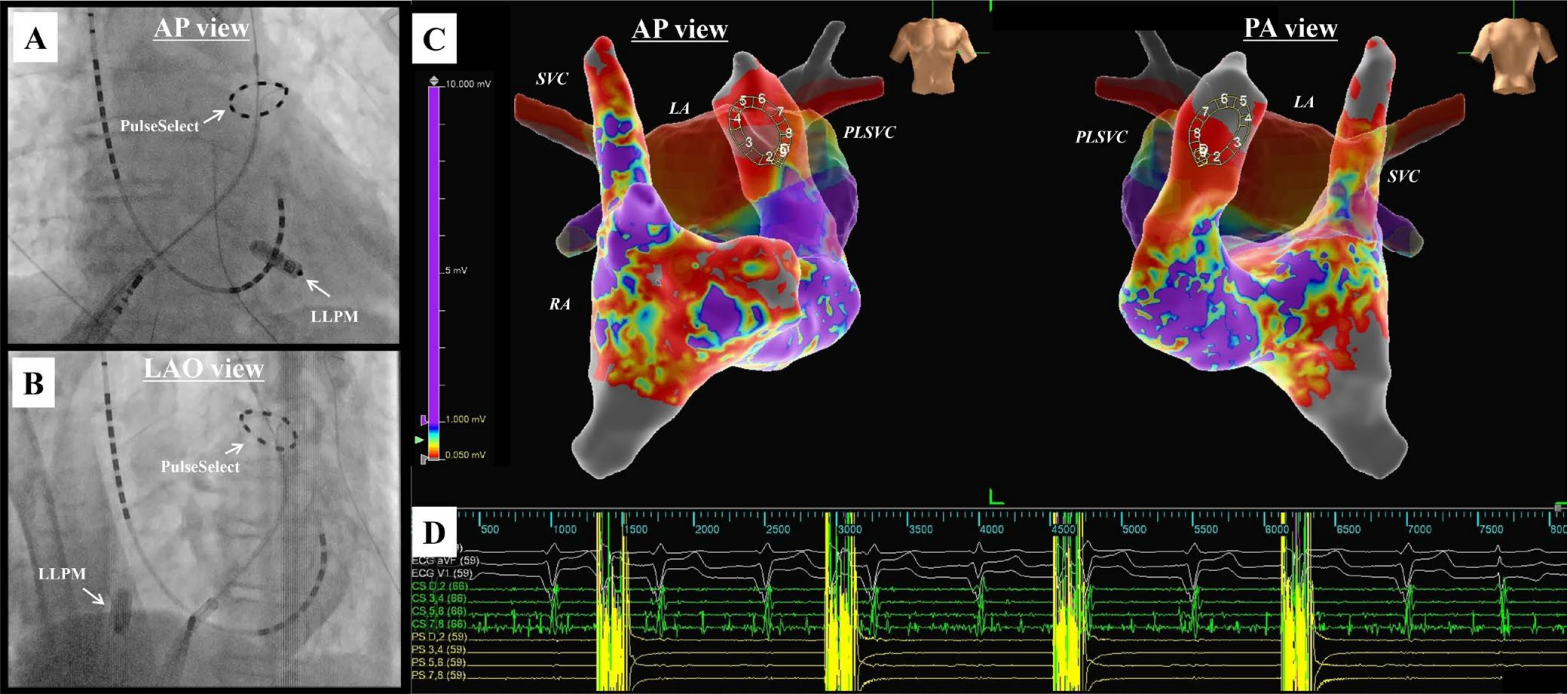
PFA for the PLSVC with LLPM. (A, B) Fluoroscopic images. (C, D) No device interference was observed during PLSVC ablation. The distance between the LLPM and the PLSVC ablation site was 72 mm.

In this patient, extensive PFA involving the PLSVC, SVC, and LAPW was completed without clinically apparent device dysfunction. PFA has demonstrated favorable safety and efficacy in the treatment of AF [1]; however, the safety of PFA in patients with cardiac implantable electronic devices (CIEDs), especially a LLPM, has not been well established.

PFA performed in close proximity to LLPM requires particular caution because of the heightened risk of EMI [2]. Ventricular undersensing with a dual‐chamber leadless pacemaker (Aveir DR, Abbott) has been reported during PFA near the right inferior pulmonary vein [3]. In contrast, we observed no interference despite extensive applications to the LAPW, SVC, and PLSVC. The localized bipolar and biphasic field generated by the PulseSelect catheter may have contributed to the absence of clinically apparent device interference; however, this hypothesis requires empirical validation. The absence of clinically apparent interference at a minimum device‐to‐ablation distance of 72 mm in this case should not be interpreted as establishing a safety threshold or as evidence of safety with other PFA platforms or in other clinical scenarios. Nevertheless, our findings highlight the necessity of continuous monitoring and post‐procedural interrogation during extensive non‐PV ablation near CIEDs. Further studies are needed to clarify the risk of device interference during PF applications for non‐PV triggers in patients with CIEDs.

## Funding

The authors have nothing to report.

## Ethics Statement

The authors have nothing to report.

## Consent

The authors have nothing to report.

## Conflicts of Interest

H.F. received lecture fees from Medtronic Japan, Abbott Medical Japan, Boston Scientific Japan, Japan Lifeline, and Daiichi Sankyo Co. Ltd. The other authors declare no conflicts of interest related to this work.

## Data Availability

The data is available on request to the corresponding author.
